# Association between plasma selenium and risk of ischemic stroke: A community-based, nested, and case-control study

**DOI:** 10.3389/fnut.2022.1001922

**Published:** 2022-11-18

**Authors:** Zhuo Wang, Shiyu Hu, Yun Song, Lishun Liu, Zhengzheng Huang, Ziyi Zhou, Yaping Wei, Tengfei Lin, Meiqing Huang, Hao Zhang, Huiyuan Guo, Yong Sun, Binyan Wang, Xianhui Qin, Xiping Xu, Feng Chi, Bohua Ren, Lijie Ren

**Affiliations:** ^1^Neurology Department of Shenzhen Second People’s Hospital/First Affiliated Hospital of Shenzhen University Health Science Center, Shenzhen, China; ^2^College of Food Science and Technology, Guangdong Ocean University, Zhanjiang, China; ^3^Key Laboratory of Precision Nutrition and Food Quality, Ministry of Education, Department of Nutrition and Health, China Agricultural University, Beijing, China; ^4^Guangdong Medical University, Guangzhou, China; ^5^Shenzhen Evergreen Medical Institute, Shenzhen, China; ^6^Graduate School at Shenzhen, Tsinghua University, Shenzhen, China; ^7^Institute of Biomedicine, Anhui Medical University, Hefei, China; ^8^AUSA Research Institute, Shenzhen AUSA Pharmed Co., Ltd., Shenzhen, China; ^9^The First People’s Hospital of Lianyungang City/The First Affiliated Hospital of Kangda College of Nanjing Medical University, Lianyungang, China; ^10^Division of Nephrology, Nanfang Hospital, Southern Medical University; National Clinical Research Center for Kidney Disease; State Key Laboratory of Organ Failure Research; Guangdong Provincial Institute of Nephrology, Guangdong Provincial Key Laboratory of Renal Failure Research, Guangzhou Regenerative Medicine and Health Guangdong Laboratory, Guangzhou, China; ^11^Shenzhen Institutes of Advanced Technology, Chinese Academy of Sciences, Shenzhen, China

**Keywords:** first ischemic stroke, hypertension, LDL-C, homocysteine, selenium

## Abstract

**Background:**

The prospective association between plasma Se and stroke risk remains inconclusive. The relationship between Se and ischemic stroke among a low circulating Se status population deserves more attention, especially for Chinese people who were a high-risk group for Se deficiency.

**Objective:**

The relationship between plasma Se concentration and ischemic stroke risk in a large-scale Chinese community-based population and any potential effect modifiers were investigated.

**Methods:**

A nested, case-control study, using data from the “China H-type Hypertension Registry Study” were conducted. A total of 1,904 first ischemic stroke cases and 1,904 controls matched for age, sex, and village were included in this study. The association between plasma Se and first ischemic stroke was evaluated by conditional logistic regression analyses.

**Results:**

The median value of plasma Se was 65.8 μg/L among total participants. Overall, a significant inverse relationship between plasma Se and first ischemic stroke risk was found (per SD increment; adjusted OR: 0.87; 95% CI: 0.80 and 0.95). Accordingly, a significantly lower risk of first ischemic stroke was found in participants in quartile 3 (65.8−<77.8 μg/L) (adjusted OR: 0.78; 95% CI: 0.63 and 0.96) and quartile 4 (≥77.8 μg/L) (adjusted OR: 0.76; 95% CI: 0.59 and 0.96), compared with those in quartile 1 (<56.0 μg/L). Furthermore, a significantly lower ischemic stroke risk was found in those with lower low-density lipoprotein cholesterol (LDL-C) levels (<3.4 vs. ≥3.4 mmol/L; *P* for interaction = 0.015) or those with lower homocysteine levels (<12.1 (median) vs. ≥12.1 μmol/L; *P* for interaction = 0.027) at baseline.

**Conclusion:**

Plasma Se was significantly inversely associated with the risk of first ischemic stroke among a large-scale Chinese community-based population (most adults with hypertension and elevated total homocysteine), especially among those with lower LDL-C and lower homocysteine levels.

## Introduction

In 2019, there were nearly four million incident stroke cases in China, which is also a major cause of death and disability, leading substantially to the disease burden (1). In fact, ischemic stroke was the main type of stroke for Chinese with a proportion of more than 70% (2, 3). The adults with hypertension were considered to be at a high-risk group of stroke, and significantly higher risks of first stroke were found in those with elevated total homocysteine (4). More than 75% of adults with hypertension have been found with elevated total homocysteine in China (5). Thus, the identification and management of novel risk factors (6), are essential for stroke prevention among this high-risk group.

Selenium (Se) is a trace element that is a required cofactor for the synthesis of specific enzymes involved in anti-inflammatory processes, such as glutathione peroxidases (7). Relatively low circulating Se status in Chinese populations has been reported (8). Higher serum Se status had been found associated with a lower risk of ischemic heart disease in a Danish population, indicating Se may play a certain role in the prevention of CVD (9). However, in meta-analysis on coronary heart disease as well as some other CVDs, the association of Se and its beneficial effects against disease incidence presented as U-shaped (10). Overall, there were limited data regarding the prospective association between plasma Se status and stroke risk, and the current evidences is inconsistent (11). Serum Se concentration was not significantly associated with stroke in a 10-year follow-up study in Finland (12). Moreover, a cohort study in China indicated a significant reverse association in the relationship between Se and hemorrhagic stroke, but not for ischemic stroke (13). Recent results based on China Stroke Primary Prevention Trial (CSPPT) found between plasma Se was inversely associated with first stroke among Chinese adults with hypertension (14). Thus, the relationship between Se and ischemic stroke among a low circulating Se status population deserves more attention, especially for Chinese people who are a high-risk group in Se deficiency.

Therefore, the prospective association between plasma Se concentrations and first ischemic stroke and its potential modifiers were evaluated among a large-scale Chinese community-based population, through a nested, case-control study.

## Materials and methods

### Study design and population

Our present study is a subset of the China H-type Hypertension Registry Study (CHHRS; URL: Unique identifier: ChiCTR1800017274)^1^, which is an ongoing community-based, observational, multicenter, real-world registry study as previously reported (15). The H-type hypertension was defined as hypertension with elevated total homocysteine (tHcy ≥ 10 μmol/L) (16). Eligible participants were those aged over 18 years with essential hypertension, and hypertension was defined as baseline seated, systolic blood pressure (SBP) ≥140 mmHg and/or seated, diastolic blood pressure (DBP) ≥90 mmHg. The exclusion criteria included those who had psychological or nervous system impairment resulting in an inability to demonstrate informed consent or were unable to be followed-up according to the study protocol. The trial consisted of two stages: Screening and recruitment, and a long-term observation follow-up period. Physical examination and clinical outcomes were recorded during follow-ups every 3 months. We used a nested, case-control study design from the population of this community-based CHHRS study of Lianyungang.

### Outcomes

The primary outcome of the current study included first ischemic stroke, first hemorrhagic stroke, and first uncertain type stroke. First stroke record information was obtained *via* the Centers for Disease Control and Prevention of Lianyungang and checked against the national health insurance system with electronic linkage to all hospitalizations or ascertained through active follow-up (15).

### Statistical analysis

For continuous variables, data are presented as median (IQR) and differences in baseline characteristics between cases and controls were compared using rank sum tests. For categorical variables, data are presented as frequency (%) and differences in baseline characteristics were evaluated by chi-square tests. The association of plasma Se with ischemic stroke was evaluated as a continuous variable per SD increment and as a categorical variable using quartiles with quartile 1 (Q1) as the reference group. Conditional logistic regression analysis was performed to assess the odds ratios (OR) and 95% confidence intervals (CI) for the association between plasma Se and first ischemic stroke. Potential effect modifications of the association between plasma Se and ischemic stroke were also performed based on previous studies (17, 18). Heterogeneity among subgroups was examined by fitting unconditional logistic regressions and presented in forest plots. Likelihood ratio tests were performed to examine the interactions between subgroups and plasma Se in first ischemic stroke risk. The indirect effect of Se on first ischemic stroke mediated through other risk factors of stroke compared with the total effect of Se on first ischemic stroke was conducted by mediation analysis, using the product of coefficients method (19).

A 2-tailed *P* < 0.05 was statistically significant in all analyses. (R software version 3.6.1)^2^ and Empower version (R) (version 3.0; X&Y Solutions, Inc., Boston, MA, USA)^3^ were used for all statistical analyses.

## Results

### Study participants and characteristics

**FIGURE 1:**
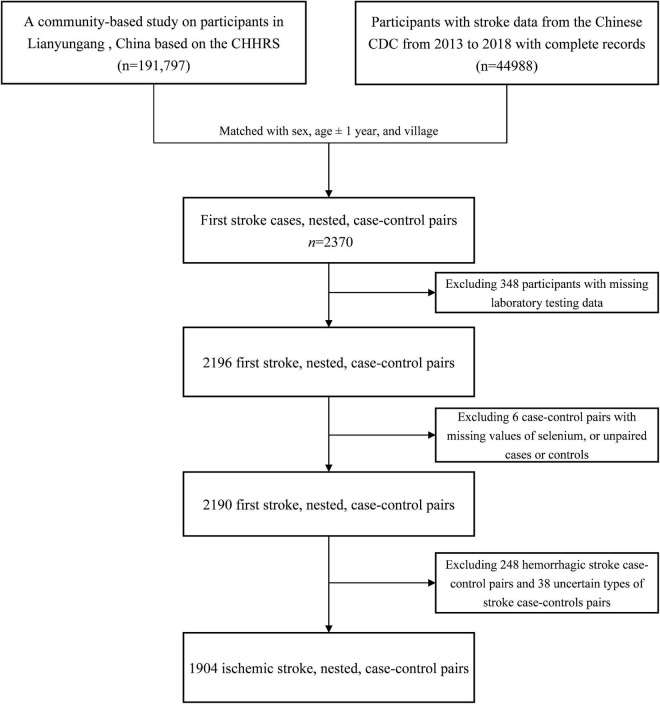
Flowchart of the study participants: A nested case-control design based on the CHHRS in Lianyungang. CHHRS, China H-type Hypertension Registry Study.

As shown in Figure 1, 1,904 ischemic stroke cases and 1,904 matched controls were included in the current study. Overall, the median of plasma Se was 65.8 μg/L among total participants. Baseline characteristics of the study participants by ischemic stroke cases and controls were presented in Table 1. Median values of plasma Se were lower in participants with stroke (65.3 μg/L) than in control participants (66.3 μg/L) and the difference was statistically significant (*P* = 0.041). Moreover, ischemic stroke cases had higher BMI, SBP, DBP, FBG, TG, LDL-C, homocysteine, and copper concentration at baseline, while HDL-C and eGFR were lower among ischemic stroke cases than that of controls. Additionally, a higher number of ischemic stroke cases had a history of hypertension, hyperlipidemia and diabetes, and use of glucose-lowering drugs and antihypertensive drugs than that of controls.

**TABLE 1:** Baseline characteristics of the study participants by ischemic stroke status.

Variables	Ischemic stroke (*n* = 1,904)	Non-stroke (*n* = 1,904)	*P*-value
Age, y†	66.5 (60.5–73.2)	66.5 (60.5–73.2)	0.994
Male, *n* (%)	767 (40.3)	767 (40.3)	1.000
BMI, kg/m^2^	25.5 (23.2–28.2)	25.2 (22.9–27.7)	<0.001
SBP, mmHg	150.3 (139.3–160.7)	147.3 (134.7–158.0)	<0.001
DBP, mmHg	91.3 (82.0–100.3)	87.3 (79.7–97.0)	<0.001
Current smoking, *n* (%)	359 (18.9)	354 (18.6)	0.835
Current alcohol drinking, *n* (%)	321 (16.9)	416 (21.8)	<0.001
**Medication use, *n* (%)**			
Antihypertensive drugs	887 (46.6)	736 (38.7)	<0.001
Glucose-lowering drugs	82 (4.3)	56 (2.9)	0.024
Lipoprotein-lowering drugs	9 (0.5)	9 (0.5)	1.000
Antiplatelet drugs	22 (1.2)	11 (0.6)	0.054
**History of diseases, *n* (%)**			
Self-reported hypertension	1,367 (71.8)	1,186 (62.3)	<0.001
Self-reported diabetes	224 (11.8)	154 (8.1)	<0.001
Self-reported hyperlipidemia	187 (9.8)	119 (6.2)	<0.001
**Laboratory results**			
Fasting glucose, mmol/L	5.7 (5.2–6.7)	5.6 (5.2–6.3)	<0.001
Total cholesterol, mmol/L	5.2 (4.5–6.0)	5.2 (4.5–6.0)	0.805
Triglycerides, mmol/L	1.4 (1.0–2.0)	1.2 (0.9–1.9)	<0.001
HDL-C, mmol/L	1.5 (1.2–1.7)	1.5 (1.3–1.8)	<0.001
LDL-C, mmol/L	3.1 (2.6–3.7)	3.0 (2.6–3.6)	0.002
Homocysteine, μmol/L	12.3 (10.2–15.2)	11.8 (9.9–14.3)	<0.001
eGFR, mL/min/1.73 m^2^	95.9 (88.4–103.6)	96.8 (89.6–103.9)	0.015
Selenium, μg/L	65.3 (55.1–77.5)	66.3 (56.7–77.8)	0.041
Retinol, μg/dL	51.2 (42.9–61.1)	51.0 (42.9–60.6)	0.437
Copper, μg/dL	101.3 (89.2–114.7)	100.8 (88.6–112.9)	0.030

^†^For continuous variables, values are median (IQR).

Differences in characteristics were compared using rank sum tests for continuous variables and chi-square tests for categorical variables.

In addition, baseline characteristics among participants, stratified by Se concentration, are illustrated in Supplementary Table 2. Participants with higher plasma Se concentrations tended to have higher BMI, SBP, DBP, FBG, TC, HDL-C, LDL-C, retinol, and copper, whereas they tended to have lower homocysteine. Participants with higher Se concentrations have a higher proportion of current smokers and alcohol drinkers and have a history of hypertension, diabetes, and hyperlipidemia and a higher proportion of antihypertensive drugs usage than those with lower Se concentrations. Besides, the baseline characteristics stratified by sex were illustrated in Supplementary Table 1.

### Association between selenium and first ischemic stroke

**FIGURE 2:**
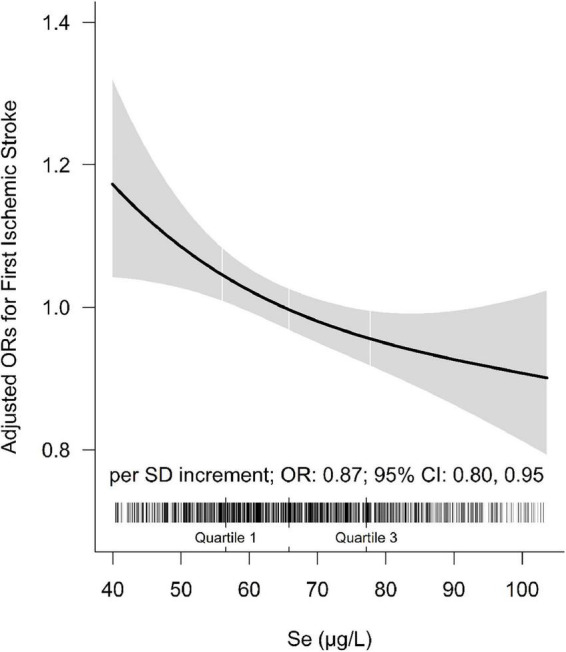
The association between baseline plasma selenium (Se) and risk of ischemic stroke. Adjusted for BMI, body mass index; smoking, drinking status, SBP, DBP, hypertension, self-reported diabetes, self-reported hyperlipidemia, fasting glucose, TC, total cholesterol; TG, triglycerides, HDL-C, high-density lipoprotein cholesterol; LDL-C, low-density lipoprotein cholesterol, tHcy, total homocysteine, eGFR, plasma retinol, and plasma copper concentration.

The association between plasma Se and ischemic stroke risk was shown in Table 2. Overall, a significant inverse association between plasma Se and the risk of first ischemic stroke (per SD increment; adjusted OR: 0.87; 95% CI: 0.80 and 0.95) was found, after adjustment for important confounders (Figure 2). Consistently, a significant, lower ischemic stroke risk was found among participants in quartile 3 (65. 8−<77.8 μg/L) and quartile 4 (≥77.8 μg/L). Specifically, the adjusted ORs of quartile 3 and quartile 4 were 0.78 (95% CI: 0.63 and 0.96) and 0.76 (95% CI: 0.59 and 0.96), respectively, when compared with those in quartile 1 (<56.0 μg/L) (*P*-trend = 0.017).

**TABLE 2:** Association of plasma selenium with first ischemic stroke*.

Selenium, μg/L	*N*	Cases (%)	Crude model	*P*-value	Adjusted model^1^	*P*-value
			OR (95%CI)		OR (95%CI)	
**First ischemic stroke**						
Per SD	3,808	1,904 (50.0)	0.89 (0.82, 0.96)	0.003	0.87 (0.80, 0.95)	0.001
** Quartiles**						
Q1 (<56.0)	952	509 (53.5)	ref		ref	
Q2 (56.0 to <65.8)	952	472 (49.6)	0.83 (0.69, 1.00)	0.054	0.85 (0.70, 1.04)	0.115
Q3 (65.8 to <77.8)	952	453 (47.6)	0.75 (0.62, 0.91)	0.004	0.78 (0.63, 0.96)	0.020
Q4 (≥77.8)	952	470 (49.4)	0.78 (0.63, 0.98)	0.029	0.76 (0.59, 0.96)	0.022
*P* for trend				0.016		0.017

*ORs of first ischemic stroke were estimated by modeling plasma selenium as a continuous variable and as quartiles using conditional logistic regression.

^1^Model was adjusted for BMI, body mass index; smoking, drinking status, SBP, DBP, hypertension, self-reported diabetes, self-reported hyperlipidemia, FBG, fasting glucose, TC, total cholesterol; TG, triglycerides; HDL-C, high-density lipoprotein cholesterol; LDL-C, low-density lipoprotein cholesterol, Thcy, total homocysteine; eGFR, plasma retinol, and plasma copper concentration.

However, as shown in Supplementary Table 3, further adjustment for history of cardiovascular diseases and the use of medications did not substantially change the significant inverse relationship between plasma Se and first ischemic stroke (per SD increment; adjusted OR: 0.86; 95% CI: 0.79 and 0.94). Moreover, only 3.94% (95% CI: 0.76 and 11.53; *P* = 0.022) and 4.52% (95% CI: 0.99 and 12.85; *P* = 0.012) of the observational relationship between Se and risk of first ischemic stroke was mediated through TC and DBP, respectively (Supplementary Table 4).

### Subgroup analyses

**FIGURE 3:**
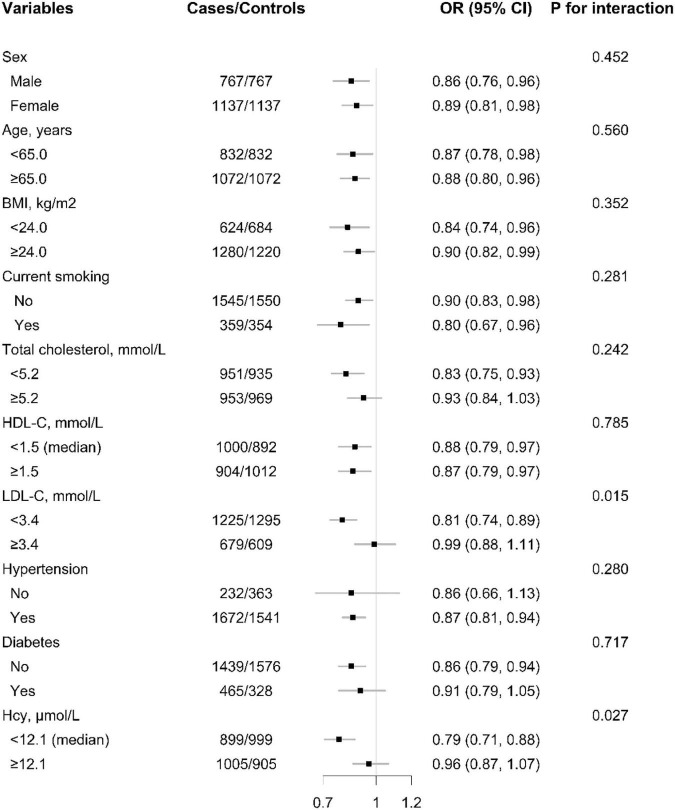
The association between baseline plasma selenium (Se) (per SD increment) concentration and risk of ischemic stroke among each subgroup. ORs of ischemic stroke in relation to plasma concentrations of selenium (Se) (per SD increment) were calculated using unconditional logistic regression models. *P*-value for interaction were calculated using log likelihood ratio tests. Each subgroup analysis adjusted, if not stratified for sex, age, BMI, body mass index; smoking, drinking status, SBP, DBP, hypertension, self-reported diabetes, self-reported hyperlipidemia, FBG, fasting glucose; TC, total cholesterol, TG, triglycerides, HDL-C, high-density lipoprotein cholesterol; LDL-C, low-density lipoprotein cholesterol; Thcy, total homocysteine, eGFR, plasma retinol, and plasma copper concentration.

The relationship of plasma Se concentration (per SD increment) with the risk of first ischemic stroke in various subgroups was examined to reveal possible modifiers (Figure 3 and Supplementary Figure 1).

A significantly lower risk of first ischemic stroke was found in those with LDL-C < 3.4 mmol/L (adjusted OR: 0.81; 95% CI: 0.74 and 0.89; vs. ≥3.4 mmol/L; adjusted OR: 0.99; 95% CI: 0.88 and 1.11; *P* for interaction = 0.015). Moreover, the inverse association between plasma Se and first ischemic stroke risk was also modified by homocysteine levels and a significantly lower ischemic stroke risk was found in participants with tHcy < 12.1 μmol/L (adjusted OR: 0.79; 95% CI: 0.71 and 0.88; vs. ≥12.1 μmol/L; adjusted OR: 0.96; 95% CI: 0.87 and 1.07; *P* for interaction = 0.027) (Figure 3).

## Discussion

The current study shows that, based on the largest nested case-control study in a Chinese population with relatively low plasma Se levels so far, a higher baseline Se concentration is associated with a lower risk of first ischemic stroke. Moreover, the inverse association of plasma Se with first ischemic stroke was more pronounced in participants with HDL-C < 3.4 mmol/L and those with tHcy < 12.1 μmol/L.

Overall, relatively limited longitudinal studies reported the association of circulating Se status with stroke risk, and their findings were still inconsistent. Based on the Chinese Dongfeng–Tongji cohort, it was found that Se was significantly negatively correlated with hemorrhagic stroke, but not with ischemic stroke (per IQR increment, adjusted OR:0.92; 95% CI: 0.82–1.05) (14). Another study found that plasma Se was significantly non-linear associated with first stroke in males (20). However, no significant association was found between serum Se and stroke in the Finnish elderly (12). It is worth noting that most previous studies have failed to exclude the traditional risk factors of stroke and the influence of other antioxidants as possible, so it may not be sufficient to fully reveal the relationship between Se and stroke risk. Especially in the Chinese population with relatively low status of Se and high-risk of Se deficiency, the evidence concerning the association between Se and stroke and its possible potential effect modifiers is still insufficient.

This study can comprehensively evaluate the dose relationship of plasma Se concentration with first ischemic stroke risk, among a large-scale Chinese community-based population with relatively low circulating Se status. Our study provides two new perspectives. First of all, this study is the largest one conducted in a Chinese community-based population with low circulating Se status so far, to evaluate the relationship between plasma Se concentration and first ischemic stroke. Plasma Se concentration was found to be inversely associated with the risk of first ischemic stroke in this study. This finding is consistent with that of a nested case-control study among a Chinese hypertensive population, which indicated first stroke and ischemic stroke risk were decreased when increasing plasma Se concentration (14). This condition might be because Se and selenoproteins are required as modulators in brain metabolism (21, 22). Glutathione peroxidase (GPX) is one of the most studied Se-dependent proteins (selenoproteins), which reduces brain damage and edema as well as inflammatory infiltration in animal models with ischemic stroke (23). Moreover, Se was considered as an antioxidant for humans, and its potential in scavenging free radicals and modulating inflammation may play a key role in lowering ischemic stroke risk (24–26). However, more research is needed to verify our findings and further examine the potential biological mechanisms.

Second, our results indicated that the relation of plasma Se with first ischemic stroke was modified by LDL-C and total homocysteine concentrations. The risk of first ischemic stroke was significantly lower among those participants with lower LDL-C concentrations (<3.4 mmol/L) and those with lower total homocysteine concentrations (<12.1 μmol/L). In our study, an increase in blood pressure, FBG levels, TC levels, and LDL-C levels corresponded to the increase of plasma Se concentrations, in keeping with the results of several previous large cross-sectional studies (27–29). Hyperlipidemia is a widely accepted risk factor of stroke, thereby Se and blood lipids may compete and inhibit each other in stroke prevention (1). Based on our data, the beneficial effect on first ischemic stroke associated with high concentrations of plasma Se may be attenuated by the adverse effect of higher LDL-C concentrations. Previous studies found interactions of serum copper, Se, and low-density lipoprotein cholesterol in atherogenesis (30). And another study indicated that *dietary Se increases cellular glutathione peroxidase activity and reduces the enhanced susceptibility to lipid peroxidation of plasma and low-density lipoprotein in kidney transplant recipients* (31). We also found that the relationship between plasma Se and first ischemic stroke was partially mediated by TC (Supplementary Table 4), which indicated the benefit of Se on ischemic stroke may be partially through the potential effect of Se on lowering TC. Overall, the interaction between plasma Se and blood lipids may play a certain role in the primary prevention of first ischemic stroke, and it still needs to be further confirmed. Additionally, the inverse relationship of plasma Se with first ischemic stroke risk was more prominent in participants with tHcy < 12.1 μmol/L in our study. This finding is consistent with the results of CSPPT (14, 32) in it was which reported that significantly lower first stroke risk was found among those with higher baseline folate concentration. *Se may mediate endothelial protective effects and thus reduce the high-risk of stroke due to hypertension and high homocysteine. A relevant study found that Se inhibits homocysteine-induced endothelial dysfunction and apoptosis via activation of AKT* (33). Thus, proper increment of dietary Se intake or Se supplementation (34–36), along with homocysteine lowering therapy (HLT) in individuals with hypertension and hyperhomocysteinaemia (Hhcy), appears to be a more acceptable strategy for stroke primary prevention based on the present study. In general, this study demonstrated that maintaining appropriate plasma Se, LDL-C and Hcy levels may be of great significance for primary prevention of stroke.

There were several limitations of this work. Firstly, although a wide range of covariates are included in the regression model, residual confounding effects of unmeasured factors cannot be excluded, so these findings should be interpreted with caution. Secondly, we only assessed baseline plasma Se concentrations for the enrolled patients. The relationship between plasma Se concentration and first ischemic stroke risk might be better illustrated if plasma Se concentration were tested at different time points during follow-up. Thirdly, since this was an analysis based on a subset study of the CHHRS, dietary information related to Se supplementation was not collected at baseline. However, dietary Se supplementation would only have a minimal impact on our population, since we enrolled people from the same study site (Lianyungang, China) who shared similar regional soil, water supply, and dietary habits. In future research, we plan to collect more detailed information on dietary Se supplementation and expand the population base to include the whole country. Fourthly, the generalization of our findings to the general population should be cautious. However, there were no differences in the relation of Se with first ischemic stroke among the normal subjects or those with hypertension in the subgroup analysis when excluding many potential confounders. Overall, our results should be considered as hypothesis generation. More research is needed to verify our results and reveal the biological mechanisms involved.

## Conclusion

We indicated that plasma Se concentrations were inversely associated with first incident ischemic stroke among an Eastern Chinese community-based population, especially in those with lower plasma LDL-C and tHcy levels. A Chinese population, especially those with hypertension and elevated total homocysteine, with well-controlled lipid and tHcy levels should be the target for future investigations on Se supplementation for the primary prevention of stroke.

## Data availability statement

The raw data supporting the conclusions of this article will be made available by the authors, without undue reservation.

## Ethics statement

The studies involving human participants were reviewed and approved by the Ethics Committee of the Institute of Biomedicine, Anhui Medical University. The patients/participants provided their written informed consent to participate in this study.

## Author contributions

ZW and SH conceived and designed the study and wrote the manuscript. YS, LL, HZ, HG, BW, XQ, YS, and XX contributed to the study concept and design. LR designed and conceptualized the study. ZZ, YW, and TL acquired the data. ZW and LL performed the data management. BR, ZW, and SH interpreted the data. MH contributed to data cleaning and methodology rechecking. All authors critical review and revision of the manuscript for important intellectual content.

## Footnotes

1
http://www.chictr.org.cn

2
www.r-project.org

3
www.empowerstats.com
