# Supplementary material for: Association between plasma selenium and risk of ischemic stroke: A community-based, nested, and case-control study
Source: Front Nutr. 2022 Nov 18;9:1001922. doi: 10.3389/fnut.2022.1001922 (PMC9716699; doi:10.3389/fnut.2022.1001922)
Supplement: Supplementary file 1 [file Data_Sheet_1.docx]

**Association between plasma selenium and risk of ischemic stroke: a community-based, nested, case-control study**

**Online Supplemental Material**

**Supplemental Table 1.** Characteristics of the study participants stratified by sex

**Supplemental Table 2.** Characteristics of the study participants according to baseline plasma selenium concentrations

**Supplemental Table 3.** Association of Plasma Selenium with ischemic stroke with further adjustment for medication usage and history of cardiovascular diseases

**Supplemental Table 4.** Causal mediation analysis between selenium and traditional stroke risk factors on the risk of first ischemic stroke

**Supplemental Figure 1.** The association between baseline plasma selenium (Se) (per SD increment) and risk of ischemic stroke among each subgroup

**Supplemental Table 1. Characteristics of the study participants stratified by sex.**

| **Variables** | **Total**  **(n=3808)** | **Male**  **(n=1534)** | **Female**  **(n=2274)** | ***P*** |
| --- | --- | --- | --- | --- |
| Age, y† | 66.5 (60.5-73.2) | 67.4 (61.3-74.0) | 65.8 (59.8-72.6) | <0.001 |
| BMI, kg/m^2^ | 25.4 (23.1-27.9) | 24.8 (22.6-27.2) | 25.7 (23.4-28.4) | <0.001 |
| SBP, mmHg | 149.0 (137.0-159.3) | 147.7 (136.0-158.3) | 150.0 (137.7-160.0) | 0.003 |
| DBP, mmHg | 89.7 (80.7-98.7) | 90.7 (81.7-100.0) | 89.0 (80.0-98.0) | <0.001 |
| Current smoking, *n* (%) | 713 (18.7) | 646 (42.1) | 67 (2.9) | <0.001 |
| Current alcohol drinking, *n* (%) | 737 (19.4) | 657 (42.8) | 80 (3.5) | <0.001 |
| **Medication use, *n* (%)** |  |  |  |  |
| Antihypertensive drugs | 1623 (42.6) | 585 (38.1) | 1038 (45.6) | <0.001 |
| Glucose-lowering drugs | 138 (3.6) | 44 (2.9) | 94 (4.1) | 0.040 |
| Lipoprotein-lowering drugs | 18 (0.5) | 5 (0.3) | 13 (0.6) | 0.278 |
| Antiplatelet drugs | 33 (0.9) | 15 (1.0) | 18 (0.8) | 0.543 |
| **History of diseases, *n* (%)** |  |  |  |  |
| Self-reported hypertension | 2553 (67.0) | 968 (63.1) | 1585 (69.7) | <0.001 |
| Self-reported diabetes | 378 (9.9) | 116 (7.6) | 262 (11.5) | <0.001 |
| Self-reported hyperlipidemia | 306 (8.0) | 93 (6.1) | 213 (9.4) | <0.001 |
| **Laboratory results** |  |  |  |  |
| Fasting glucose, mmol/L | 5.7 (5.2-6.4) | 5.7 (5.2-6.4) | 5.6 (5.2-6.5) | 0.338 |
| Total cholesterol, mmol/L | 5.2 (4.5-6.0) | 5.0 (4.3-5.7) | 5.4 (4.7-6.2) | <0.001 |
| Triglycerides, mmol/L | 1.3 (0.9-1.9) | 1.1 (0.8-1.7) | 1.4 (1.0-2.1) | <0.001 |
| HDL-C, mmol/L | 1.5 (1.3-1.8) | 1.5 (1.2-1.7) | 1.5 (1.3-1.8) | <0.001 |
| LDL-C, mmol/L | 3.1 (2.6-3.6) | 2.9 (2.5-3.4) | 3.2 (2.7-3.8) | <0.001 |
| Homocysteine, *μ*mol/L | 12.1 (10.0-14.8) | 13.3 (11.2-17.0) | 11.2 (9.4-13.5) | <0.001 |
| eGFR, mL / min /1.73 m^2^ | 96.2 (88.9-103.8) | 95.7 (88.1-103.4) | 96.7 (89.6-103.8) | 0.003 |
| Selenium, *μ*g/L | 65.8 (56.0-77.7) | 66.7 (57.4-79.0) | 65.1 (54.9-77.0) | <0.001 |
| Retinol, *μ*g/dL | 51.1 (42.9-60.8) | 55.9 (46.5-67.2) | 48.7 (41.2-57.0) | <0.001 |
| Copper, *μ*g/dL | 101.1 (88.8-113.9) | 93.9 (82.7-106.0) | 105.5 (94.3-117.2) | <0.001 |

^†^ For continuous variables, values are median (IQR).

Differences in characteristics were compared using rank sum tests for continuous variables and chi-square tests for categorical variables.

**Supplemental Table 2. Characteristics of the study participants according to baseline plasma selenium concentrations ^1^.**

| **Variables** | **Selenium *μ*g/L** | | | | |
| --- | --- | --- | --- | --- | --- |
|  | **Q1**  **(<56.0)** | **Q2**  **(56.0 to <65.8)** | **Q3**  **(65.8 to <77.8)** | **Q4**  **(≥77.8)** | ***P*** |
| **N** | 952 | 952 | 952 | 952 |  |
| Age, y^†^ | 66.7 (60.9-73.2) | 66.4 (60.5-72.7) | 66.0 (60.2-72.9) | 66.8 (60.3-73.9) | 0.363 |
| Male, *n* (%) | 333 (35.0) | 391 (41.1) | 390 (41.0) | 420 (44.1) | <0.001 |
| BMI, kg/m^2^ | 25.1 (22.7-27.4) | 25.3 (22.9-27.8) | 25.5 (23.4-28.4) | 25.6 (23.4-28.1) | <0.001 |
| SBP, mmHg | 145.3 (131.3-155.0) | 146.7 (133.9-156.8) | 149.7 (138.6-160.7) | 154.0 (145.2-165.0) | <0.001 |
| DBP, mmHg | 87.7 (77.7-98.3) | 88.7 (80.0-98.7) | 89.7 (81.3-98.3) | 91.7 (83.3-99.7) | <0.001 |
| Current smoking, *n* (%) | 153 (16.1) | 170 (17.9) | 178 (18.7) | 212 (22.3) | 0.005 |
| Current alcohol drinking, *n* (%) | 128 (13.4) | 184 (19.3) | 207 (21.7) | 218 (22.9) | <0.001 |
| **Medication use, *n* (%)** |  |  |  |  |  |
| Antihypertensive drugs | 390 (41.0) | 403 (42.3) | 384 (40.3) | 446 (46.8) | 0.018 |
| Glucose-lowering drugs | 36 (3.8) | 29 (3.0) | 37 (3.9) | 36 (3.8) | 0.745 |
| Lipoprotein-lowering drugs | 4 (0.4) | 2 (0.2) | 4 (0.4) | 8 (0.8) | 0.236 |
| Antiplatelet drugs | 6 (0.6) | 8 (0.8) | 9 (0.9) | 10 (1.1) | 0.784 |
| **History of diseases, *n* (%)** |  |  |  |  |  |
| Self-reported hypertension | 587 (61.7) | 633 (66.5) | 628 (66.0) | 705 (74.1) | <0.001 |
| Self-reported diabetes | 82 (8.6) | 71 (7.5) | 99 (10.4) | 126 (13.2) | <0.001 |
| Self-reported hyperlipidemia | 68 (7.1) | 54 (5.7) | 79 (8.3) | 105 (11.0) | <0.001 |
| **Laboratory results** |  |  |  |  |  |
| Fasting glucose, mmol/L | 5.5 (5.2-6.1) | 5.6 (5.2-6.3) | 5.7 (5.2-6.5) | 5.8 (5.2-6.9) | <0.001 |
| Total cholesterol, mmol/L | 4.9 (4.2-5.5) | 5.1 (4.5-5.9) | 5.4 (4.6-6.1) | 5.6 (4.9-6.5) | <0.001 |
| Triglycerides, mmol/L | 1.3 (0.9-1.9) | 1.3 (0.9-1.9) | 1.2 (0.9-1.8) | 1.3 (0.9-2.0) | 0.319 |
| HDL-C, mmol/L | 1.4 (1.2-1.6) | 1.5 (1.3-1.7) | 1.6 (1.3-1.8) | 1.6 (1.3-1.9) | <0.001 |
| LDL-C, mmol/L | 2.9 (2.4-3.4) | 3.0 (2.6-3.5) | 3.1 (2.6-3.7) | 3.3 (2.8-3.9) | <0.001 |
| Homocysteine, *μ*mol/L | 12.7 (10.5-15.7) | 12.1 (10.1-14.6) | 11.7 (9.8-14.2) | 11.6 (9.7-14.3) | <0.001 |
| eGFR, mL · min^−1^ · 1.73 m^−2^ | 96.0 (89.3-103.6) | 96.8 (89.6-103.6) | 96.4 (89.1-104.2) | 95.9 (88.1-103.4) | 0.207 |
| Selenium, *μ*g/L | 49.6 (44.8-52.9) | 61.0 (58.4-63.6) | 71.3 (68.5-74.3) | 87.4 (81.9-95.1) | <0.001 |
| Retinol, *μ*g/dL | 47.2 (39.4-55.8) | 49.9 (42.3-59.0) | 51.9 (43.8-61.5) | 55.9 (47.3-66.9) | <0.001 |
| Copper, *μ*g/dL | 99.7 (86.9-112.4) | 101.1 (88.9-113.1) | 101.1 (90.1-113.9) | 102.1 (90.8-115.0) | 0.004 |

^1^ For continuous variables, values are median (IQR).

^†^ Differences in characteristics were compared using chi-square tests for categorical variables and rank sum tests for continuous variables.

**Supplemental Table 3. Association of Plasma Selenium with ischemic stroke with further adjustment for medication usage and history of cardiovascular diseases ^*^.**

| **Selenium, *μ*g/L** | **N** | **Cases (%)** | **Crude model** | ***P*** | **Adjusted model ^1^** | ***P*** |
| --- | --- | --- | --- | --- | --- | --- |
|  |  |  | **OR (95%CI)** |  | **OR (95%CI)** |  |
| **First ischemic stroke** |  |  |  |  |  |  |
| **Per SD** | 3808 | 1904 (50.0) | 0.89 (0.82, 0.96) | 0.003 | 0.86 (0.79, 0.94) | 0.001 |
| **Quartiles** |  |  |  |  |  |  |
| Q1 (<56.0) | 952 | 509 (53.5) | ref |  | ref |  |
| Q2 (56.0 to <65.8) | 952 | 472 (49.6) | 0.83 (0.69, 1.00) | 0.054 | 0.85 (0.70, 1.04) | 0.120 |
| Q3 (65.8 to <77.8) | 952 | 453 (47.6) | 0.75 (0.62, 0.91) | 0.004 | 0.78 (0.63, 0.96) | 0.021 |
| Q4 (≥77.8) | 952 | 470 (49.4) | 0.78 (0.63, 0.98) | 0.029 | 0.74 (0.58, 0.95) | 0.017 |
| *P* for trend |  |  |  | 0.016 |  | 0.013 |

^*^ ORs of first ischemic stroke were estimated by modeling plasma selenium as a continuous variable and as quartiles using conditional logistic regression.

^1^ Model was adjusted for body mass index (BMI), smoking, drinking status, SBP, DBP, hypertension, self-reported diabetes, self-reported hyperlipidemia, fasting glucose (FBG), total cholesterol (TC), triglycerides (TG), high-density lipoprotein cholesterol (HDL-C), low-density lipoprotein cholesterol (LDL-C), total homocysteine (tHcy), eGFR, plasma retinol, plasma copper concentration, antihypertensive drugs, glucose-lowering drugs, lipoprotein-lowering drugs, antiplatelet drugs, and history of coronary heart disease, atrial fibrillation, and heart failure.

**Supplemental Table 4. Causal mediation analysis between selenium and traditional stroke risk factors on the risk of first ischemic stroke ^*^**

| Variables | Estimate | 95% CI lower^1^ | 95% CI upper | *P* value |
| --- | --- | --- | --- | --- |
| **Total cholesterol** |  |  |  |  |
| Total effect | - 0.0375 | - 0.0565 | - 0.0173 | <0.001 |
| Mediation effect | -0.0015 | -0.0032 | -0.0003 | 0.022 |
| Direct effect | - 0.0360 | - 0.0554 | - 0.0156 | <0.001 |
| Proportion mediated | 0.0394 | 0.0076 | 0.1153 | 0.022 |
| **Triglycerides** |  |  |  |  |
| Total effect | -0.0368 | -0.0549 | -0.0161 | <0.001 |
| Mediation effect | -0.0001 | -0.0008 | 0.0018 | 0.882 |
| Direct effect | -0.0367 | -0.0555 | -0.0164 | <0.001 |
| Proportion mediated | 0.0014 | -0.0609 | 0.0259 | 0.882 |
| **HDL-C** |  |  |  |  |
| Total effect | -0.0366 | -0.0555 | -0.0165 | <0.001 |
| Mediation effect | 0.0002 | -0.0006 | 0.0009 | 0.752 |
| Direct effect | -0.0367 | -0.0556 | -0.0165 | <0.001 |
| Proportion mediated | -0.0041 | -0.0321 | 0.0182 | 0.752 |
| **LDL-C** |  |  |  |  |
| Total effect | -0.0376 | -0.0567 | -0.0174 | <0.001 |
| Mediation effect | -0.0008 | -0.0021 | 0.0001 | 0.096 |
| Direct effect | -0.0367 | -0.0560 | -0.0164 | <0.001 |
| Proportion mediated | 0.0220 | -0.0036 | 0.0685 | 0.096 |
| **SBP** |  |  |  |  |
| Total effect | -0.0355 | -0.0551 | -0.0151 | <0.001 |
| Mediation effect | 0.0013 | -0.0008 | 0.0040 | 0.236 |
| Direct effect | -0.0367 | -0.0556 | -0.0169 | <0.001 |
| Proportion mediated | -0.0356 | -0.1660 | 0.0242 | 0.236 |
| **DBP** |  |  |  |  |
| Total effect | -0.0385 | -0.0576 | -0.0184 | <0.001 |
| Mediation effect | -0.0017 | -0.0036 | -0.0004 | 0.012 |
| Direct effect | -0.0367 | -0.0554 | -0.0165 | <0.001 |
| Proportion mediated | 0.0452 | 0.0099 | 0.1285 | 0.012 |
| **Total homocysteine** |  |  |  |  |
| Total effect | -0.0397 | -0.0592 | -0.0205 | <0.001 |
| Mediation effect | -0.0051 | -0.0155 | 0.0011 | 0.080 |
| Direct effect | -0.0346 | -0.0528 | -0.0143 | <0.001 |
| Proportion mediated | 0.1285 | -0.0243 | 0.4066 | 0.080 |

^*^ Generalized additive (GAM) model was used to smooth mediator effect on outcome

^1^ Nonparametric Bootstrap (resamples=1000) Confidence Intervals with the Percentile Method

Except for the variable be analyzed, model was adjusted for the age, sex, body mass index (BMI), hypertension, self-reported diabetes, self-reported hyperlipidemia, smoking, drinking status, systolic blood pressure (SBP), diastolic blood pressure (DBP), fasting blood glucose (FBG), total cholesterol (TC), triglycerides (TG), high density lipoprotein cholesterol (HDL-C), low density lipoprotein cholesterol (LDL-C), total homocysteine (tHcy), estimated glomerular filtration rate (eGFR), plasma retinol and plasma copper.


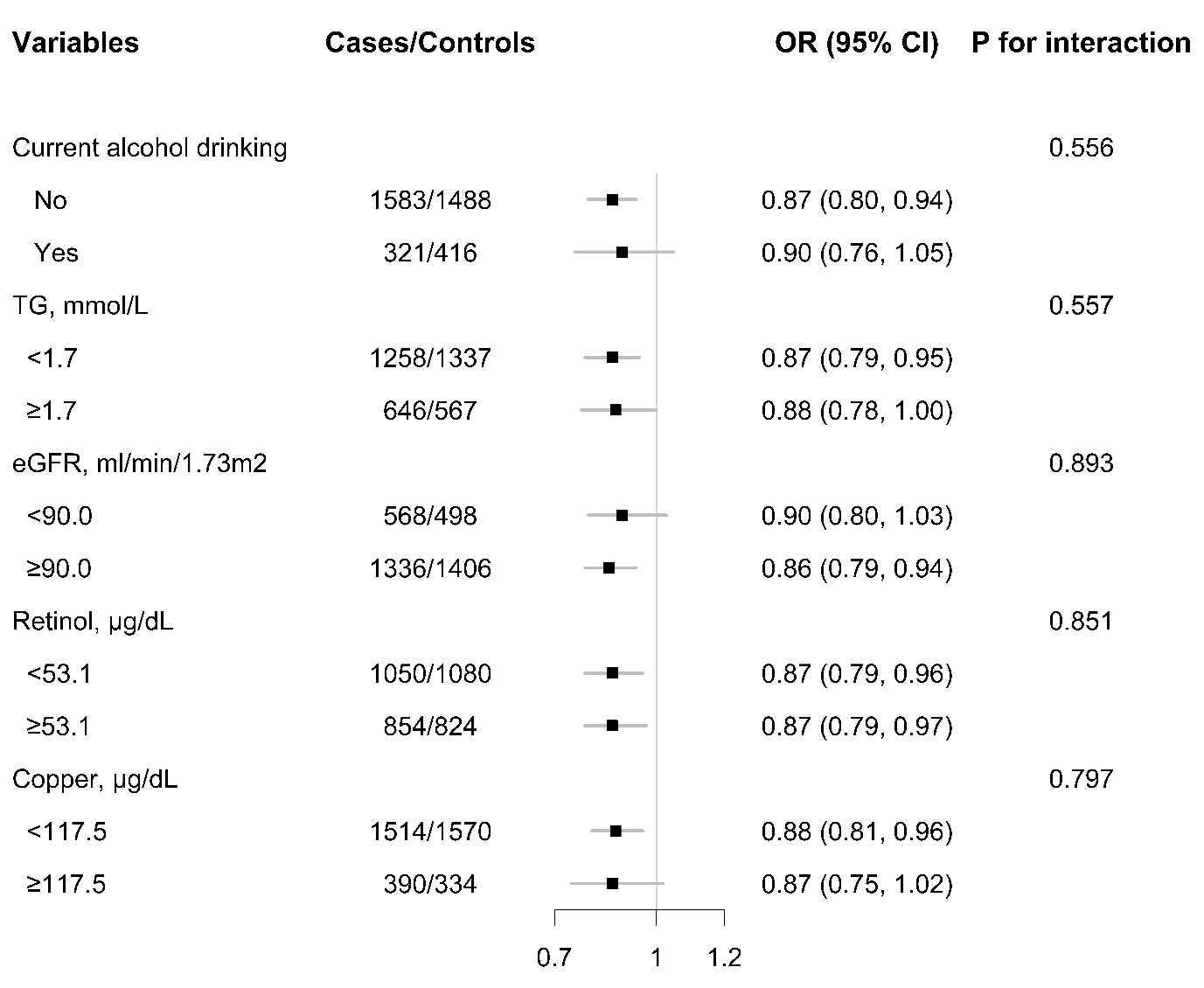


**Supplemental Figure 1. The association between baseline plasma selenium (Se) (per SD increment) and risk of ischemic stroke among each subgroup.** ORs of ischemic stroke in relation to plasma concentrations of selenium (Se) (per SD increment) were calculated using unconditional logistic regression models. *P* value for interaction were calculated using log likelihood ratio tests. Each subgroup analysis adjusted, if not stratified for sex, age, body mass index (BMI), smoking, drinking status, SBP, DBP, hypertension, self-reported diabetes, self-reported hyperlipidemia, fasting glucose (FBG), total cholesterol (TC), triglycerides (TG), high-density lipoprotein cholesterol (HDL-C), low-density lipoprotein cholesterol (LDL-C), total homocysteine (tHcy), eGFR, plasma retinol and plasma copper concentration.
